# Response of Dermal Fibroblasts to Biochemical and Physical Cues in Aligned Polycaprolactone/Silk Fibroin Nanofiber Scaffolds for Application in Tendon Tissue Engineering

**DOI:** 10.3390/nano7080219

**Published:** 2017-08-11

**Authors:** Chih-Hao Chen, Shih-Hsien Chen, Chang-Yi Kuo, Meng-Lun Li, Jyh-Ping Chen

**Affiliations:** 1 Department of Chemical and Materials Engineering, Chang Gung University, Taoyuan 33302, Taiwan; chchen5027@gmail.com (C.-H.C.); e26449@hotmail.com (S.-H.C.); onesky1997@hotmail.com (C.-Y.K.); lujason45k13@hotmail.com (M.-L.L.); 2 Department of Plastic and Reconstructive Surgery and Craniofacial Research Center, Chang Gung Memorial Hospital, Kwei-San, Taoyuan 33305, Taiwan; 3 Institute Research Center for Chinese Herbal Medicine and Research Center for Food and Cosmetic Safety, College of Human Ecology, Chang Gung University of Science and Technology, Kwei-San, Taoyuan 33302, Taiwan; 4 Department of Materials Engineering, Ming Chi University of Technology, Tai-Shan, New Taipei City 24301, Taiwan

**Keywords:** silk fibroin, polycaprolactone, nanofibers, alignment, tendon, tissue engineering

## Abstract

Silk fibroin (SF) and fiber alignment were introduced into polycaprolactone (PCL)-based electrospun nanofibers as chemical and physical cues for tendon tissue engineering applications. The physicochemical properties of random PCL (RP) nanofibers, random PCL/SF (RPSF) nanofibers and aligned PCL/SF (APSF) nanofibers were characterized for fiber orientation and SF blending effects. An in vitro cell culture with rabbit dermal fibroblasts (RDFBs) on nanofibers indicated that SF promotes cell proliferation to a higher extent than fiber alignment. Cells aligned in the direction of fiber axes could be confirmed through scanning electron microscopy (SEM) observation and cytoskeleton staining. The quantitative real-time polymerase chain reaction (qRT-PCR) experiments indicated up-regulated gene expression of tendon marker proteins (type I collagen (Col I), fibronectin and biglycan) on APSF nanofibers and tendon reconstruction was confirmed from Col III gene expression. Animal experiments with Achilles tendon defect repairs in rabbits were carried out with RPSF and APSF scaffolds. The beneficial effects of fiber alignment were verified from histological and immunohistochemical staining, where cell migration and extracellular matrix protein deposition tend to stretch in a parallel direction along the axial direction of APSF nanofibers with enhanced Col I and tenascin C production. Biomechanical testing indicated the tensile stiffness and maximum load of cell-seeded APSF scaffolds were 60.2 and 81.3% of normal tendon values, respectively, which are significantly higher than cell-seeded RPSF or acellular APSF and RPSF scaffolds. These results suggest that APSF nanofiber scaffolds combined with RDFBs have the potential to repair the gap defects of Achilles tendons in vivo and to effectively restore the function and structure of tendons.

## 1. Introduction

Due to the unique structure and functions of tendons, the repair of tendon defects remains a major challenge for clinicians. Ineffective treatments of patients frequently lead to the functional impairment of tendons. In addition, hypocellularity and hypovascularity of tendons reduce the healing ability; thus, full recovery is relatively difficult [1]. Current tendon defect reconstruction or repair is based on artificial and autologous tendons or allografts. Since allografts have several limitations such as the risk of transmitting infectious diseases, a lack of durability and functionality, insufficient supply, a potential incompatibility with the recipient’s body and immune system and the difficulty of their preservation during transportation, the autograft method is more widely used in clinical practices [2,3,4]. Although autografts may produce satisfactory long-term results and be compatible with the body’s immune system, patients often experience donor site morbidities. Moreover, the grafts are unable to achieve the required biomechanical strength during the tendon reconstruction process due to poor graft efficiency [5,6]. On the other hand, tissue-engineered scaffolds of varying sizes and shapes can be combined with special physical and biochemical cues to provide a suitable environment for cells to attach and proliferate in order to facilitate matrix deposition in scaffolds, and thus to induce tendon regeneration and accelerate tendon repair [7,8,9,10].

Electrospinning can easily and directly process biocompatible polymers into nanofibers. The nanofibrous scaffold will have a high surface area and proper pore size that can be applied for various applications, such as drug delivery, wound dressing and tissue engineering [11,12]. However, typical nanofibers that have been fabricated via electrospinning were usually collected in a random orientation that exhibits isotropic mechanical properties. In order to further enhance the mechanical strength and possibly provide physical cues for attached cells, methods have been proposed to induce the aligned orientation of electrospun nanofibers [13]. The anisotropic structure of nanofibers will be suitable for tendons, ligament, muscle and nerve tissue engineering as an ideal scaffold [4]. Specifically, with the unique anisotropic structure and biomechanical property of tendon tissue, the ideal scaffold for tendon tissue engineering should have an analogous form and possess comparable physical properties that would enhance the quality of engineered tendon tissue. Aligned nanofibers thus represent a unique group of fibrous scaffolds that not only resemble the anisotropic structure but also induce a particular cell orientation response [4].

Polycaprolactone (PCL) is a semi-crystalline, biocompatible and biodegradable polymer with a melting point of 60 °C and a glass transition temperature of −60 °C. Several reports have demonstrated PCL as an ideal scaffold material for tendon tissue engineering due to its good mechanical properties [13]. However, the hydrophobic property and slow degradation of PCL may limit its application in tissue engineering. As one of the two proteins secreted by silk worms, silk fibroin (SF) could be used as for various biomedical applications [14]. Existing literature supports the notion that SF, as a matrix material, can promote cell growth [15,16]. With its minimal inflammatory response in vivo and high oxygen/water permeability, SF could be deemed one of the best biomaterials for skeletal tissue regeneration [17]. Scaffolds fabricated from SF were applied for different regenerative medicine applications to regenerate bone, eye, nerve, skin, tendon, ligament, and cartilage [18]. Together with its hydrophilic property, we postulated that SF, acting as biochemical cues, could be blended with PCL to fabricate nanofibers to improve the cellular response of nanofiber scaffolds. 

Type I collagen (Col I) fiber bundles are major extracellular matrix components of tendons, in addition to a small amount of other types of collagens and matrix materials. Being oriented parallel to their long axes, Col I fiber bundles endow tendons with the ability to resist high tensile loads. Tendons also contain various types of cells (mainly fibroblasts) that are arranged in a parallel manner [19]. Therefore, aligned nanofibers that can support fibroblast attachment and guide cell proliferation along the co-axial orientation of nanofibers, combined with subsequent extracellular matrix (ECM) secretion with a controlled parallel spatial deposit of ECM proteins, will be a preferred scaffold for tendon tissue engineering [20]. 

Combining biochemical cues from SF and physical cues from fiber alignment, aligned PCL/SF nanofibers constitute a desirable nanofiber scaffold for tendon tissue engineering. By combining the favorable properties of PCL and SF through their use in the process of fabricating a new, blended, aligned nanofiber scaffold, we will demonstrate that the cells/scaffold construct from rabbit dermal fibroblasts (RDFBs) and the aligned PCL/SF nanofiber scaffold are suitable for repairing Achilles tendon defects in rabbit/animal model.

In this study, SF and PCL were used as basal materials to fabricate random PCL (RP) nanofibers, random PCL/SF (RPSF) nanofibers and aligned PCL/SF (APSF) nanofibers through electrospinning. The physicochemical properties of nanofiber scaffolds were first characterized in detail, followed by in vitro cell culture with rabbit dermal fibroblasts (RDFBs) to compare the cellular response of RDFBs to nanofibers with biochemical and physical cues. The nanofiber scaffolds were lastly combined with RDFBs to examine the feasibility of Achilles tendon defect repair in rabbits.

## 2. Results and Discussion

### 2.1. Characterization of Nanofibers

#### 2.1.1. SEM Analysis

**Figure 1:**
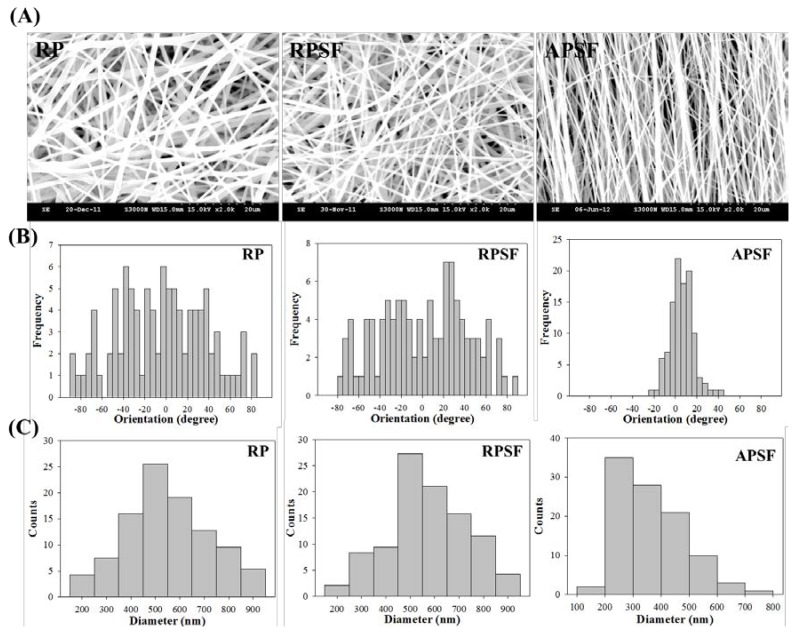
(**A**) Scanning electron microscope (SEM) analysis of random polycaprolactone (PCL) (RP) nanofibers, random PCL/silk fibroin (SF) (RPSF) nanofibers and aligned PCL/SF (APSF) nanofibers. From SEM analysis, the histograms representing fiber orientation (relative to a vertical line setting at 0°); (**B**) and fiber diameter distribution; (**C**) were calculated.

Figure 1A shows the SEM images of RP, RPSF and APSF nanofibers. The images confirm no bead formation on the surface of the nanofibers. By using static and rotating collectors, RP and RPSF nanofiber scaffolds are expected to be composed of random nanofibers, while the APSF nanofiber scaffold will contain aligned nanofibers. Indeed, significant fiber orientation differences between RP (RPSF) and APSF could be observed, with APSF displaying a uniform aligned nanofiber arrangement in one direction. It has been shown that cells were guided to align along the topographical alignment of aligned nanofibers and fiber alignment had a profound effect on cell proliferation, cell orientation and tendon strength [21]. The hydrophilicity of the nanofiber scaffold was determined by measuring the water contact angles, as shown in the inserts of Figure 1A. The RP showed a water contact angle of 117.5°. However, by adding SF, the water contact angle of the RPSF was significantly reduced to 91.4°, indicating the formation of a comparatively more hydrophilic surface. We hypothesized that the increased hydrophilic nature of RPSF is due to the blending with SF, which contained a significant number of hydrophilic functional groups and thus improved the hydrophilicity of RPSF. In comparison, the APSF exhibited a more significant drop in water contact angle to 27.5° when the fiber orientation was changed from random to alignment. According to the literature, the surface roughness of materials can create higher degrees of barrier effect, which would prevent the wider extension of water droplets on the substrate surface, and thereby increase the water contact angle [22]. Since APSF will have a smoother surface, a lower degree of roughness with an axial alignment of nanofibers, its water contact angle is much lower than that of the randomly oriented RPSF [23]. The physical effect (due to fiber orientation) on reducing the water contact angle could be seen to be much more pronounced than the biochemical effect (by blending PCL with SF). 

The orientation of nanofibers is shown in Figure 1B with histograms showing the fiber orientation of RP, RPSF, and APSF nanofibers relative to a vertical line setting at 0°. The angular distribution of RP (41.2°), RPSF (43.6°), and APSF (14.2°) indicates APSF has a significant alignment compared to RP and RPSF. Figure 1C shows the diameter distribution of nanofibers. The average fiber diameter of RP (mean ± standard deviation (SD)) is 549.4 ± 172.4 nm, which is similar to that of RPSF at 582.5 ± 178.6 nm. Although the fiber diameter of APSF appeared to shift to a lower value (363.7 ± 116.0 nm), there is no significant difference of the average fiber diameter among all nanofiber scaffolds. This might be due to the insufficient number of measurements of fiber diameters.

#### 2.1.2. Thermogravimetric Analysis (TGA) and Derivative Thermogravimetric Analysis (DTA)

**Figure 2:**
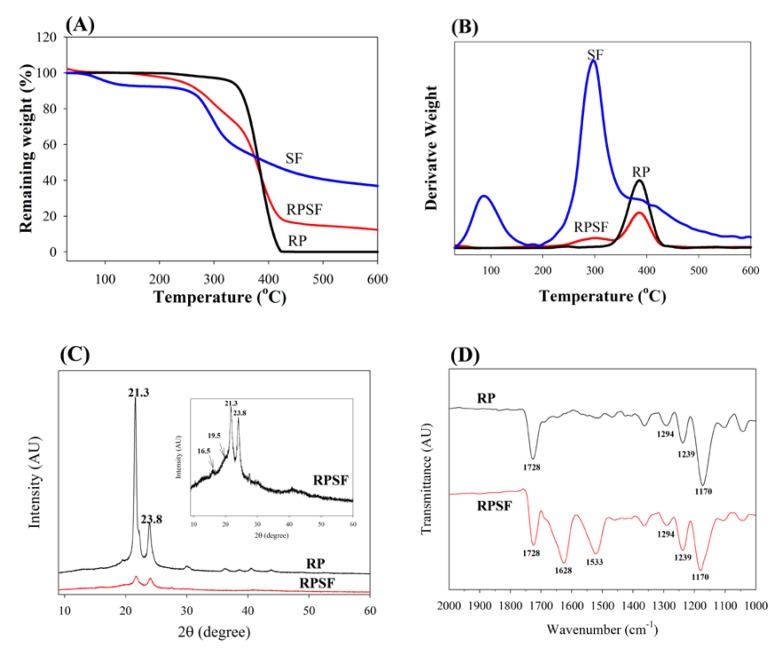
TGA (**A**) and DTA (**B**) of random PCL (RP) and random PCL/SF (RPSF) nanofiber scaffolds and SF sponges. XRD (**C**) and FTIR (**D**) analysis of random PCL (RP) and random PCL/SF (RPSF) nanofiber scaffolds.

To determine the thermal properties, TGA was used to obtain the decomposition curves of RP and RPSF nanofiber scaffolds, and to compare these with that of SF sponge. Since the thermal decomposition curve of SF sponge is similar to that of SF nanofibers [24], SF sponge was used for comparison here. The TGA results in Figure 2A shows that the decomposition of RP started at ~350 °C due to PCL pyrolysis and the residual weight approached zero after 420 °C, as usually observed for a synthetic polymer [25]. From the DTA results in Figure 2B, RP exhibited a peak thermal decomposition temperature at 380 °C. In comparison, the SF sponge began to show a significant weight loss when the temperature reached 250 °C (Figure 2A) and the peak temperature was at 300 °C (Figure 2B). Since SF has a comparatively more complex protein structure, the pyrolysis process remained incomplete until 600 °C and the percentage of weight retained in SF at 600 °C was ~35% of the initial weight. This could be attributed to the slow thermal decomposition nature of natural polymers compared to synthetic ones [26]. The initiation decomposition of SF at ~100°C was due to the presence of moisture. RPSF showed an intermediate residual weight at 600 °C (~14%) due to blending PCL with SF. Most importantly, the DTA curves of RPSF showed two peak temperatures corresponding to those of SF and RP at 300 and 380 °C, respectively, confirming the blending of PCL with SF, and each polymer in composite PCL/SF nanofibers did not influence the thermal properties of the other polymer. 

#### 2.1.3. XRD and FTIR Analysis

XRD was employed to investigate the crystal lattice in the electrospun nanofibers (Figure 2C). Both RP and RPSF nanofibers displayed (110) and (200) diffraction peaks of PCL at 21.3° and 23.7°. Two additional weak peaks could be identified in RPSF as methanol-treated SF would exhibit a major diffraction peak at 19.5° and two minor peaks at 16.5° and 22.5°, corresponding to the 4.55, 5.37, and 3.98 Å spacing, respectively, which are characteristics of the β-sheet crystalline structure [27]. The last SF peak at 22.5° was merged with the strong PCL peak at 21.3°. Therefore, RPSF shows diffraction peaks from PCL, with a domination of PCL over SF in determining the crystalline structure of nanofibers. However, the intensities of the (110) and (200) diffraction peaks of the RP were greater than those of RPSF, indicating that SF may interfere with the development of PCL crystals in RPSF [28]. The presence of SF in the PCL/SF blend will therefore influence the crystalline structure of PCL from XRD analysis. 

The FTIR spectra confirmed the blending of SF with PCL (Figure 2D). Typical vibration peaks associated with PCL were observed in the spectra of RP. These include 1728 cm^−1^ (carbonyl C=O stretching), 1294 cm^−1^ (C–O and C–C stretching), 1239 cm^−1^ (asymmetric C–O–C stretching) and 1170 cm^−1^ (symmetric C–O–C stretching) [29]. The characteristic transmittance peaks of RPSF show both SF and PCL characteristic peaks. Additional transmittance peaks of SF in RPSF compared with RP can be identified at 1628 cm^−1^ (amide I) and 1533 cm^−1^ (amide II), with 1241 cm^−1^ (amide III) merged with the C–O–C asymmetric stretching band of PCL in the composite. The shift of the transmittance peaks from 1650 cm^−1^ (amide I) and 1538 cm^−1^ (amide II), which represents a random coil conformation [30], to 1628 and 1533 cm^−1^ also indicates the ethanol post-treatment step successfully insolubilized SF with a transition of SF from the random coil to β-sheet conformation.

#### 2.1.4. Mechanical Test

**Figure 3:**
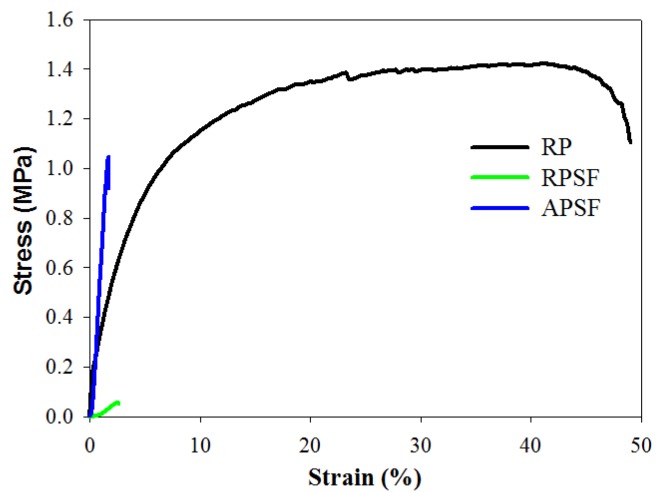
The tensile stress-strain curves of random PCL (RP), random PCL/SF (RPSF) and aligned PCL/SF (APSF) nanofiber scaffolds.

The effect of fiber alignment and SF blending on the mechanical properties of nanofiber scaffolds was studied with random and aligned scaffolds of roughly the same thickness (~100 μm). Table 1 and Figure 3 show the favorable mechanical strength and ductility of RP over RPSF scaffolds. A previous study indicated that PCL is subject to acidic hydrolysis by trifluoroacetic acid (TFA) over time, which might decrease the mechanical properties of RPSF vs. RP [31]. Comparing RPSF and APSF, although the ultimate strain is comparable, the Young’s modulus and the ultimate stress were significantly higher for the aligned nanofiber than the random nanofiber scaffolds. The ultimate stress increased 16-fold and the modulus increased 18-fold when random fiber morphology was changed to aligned fiber morphology. The Young’s modulus of APSF is also significant higher than that of RP (4.6-fold). Indeed, previous reports showed that the mechanical properties of nanofiber scaffolds are highly dependent on the orientation distribution and the components of electrospun nanofibers [32,33]. Nonetheless, the large increase in mechanical properties for the aligned scaffold may be also influenced by the higher packing factor of aligned fibers when compared to randomly-oriented fibers. Thus, we could not rule out the possibility that more fibers per unit scaffold volume may be the cause of the remarkable increase in the mechanical performance of APSF. As discussed before, the XRD analysis indicates SF may interfere with the development of PCL crystals in RPSF (Figure 2C), which may further influence the mechanical properties after blending PCL with SF in RPSF. Although SF does not result in improved mechanical properties, enhanced cellular response may be possible by introducing SF in RPSF (will be shown below). Nonetheless, we could verify that the aligned structural arrangement of nanofibers engendered a much higher mechanical strength than random nanofibers, which would contribute to the generation of a more robust scaffold for tendon tissue engineering.

### 2.2. In Vitro Cell Culture

#### 2.2.1. Cell Proliferation

**Figure 4:**
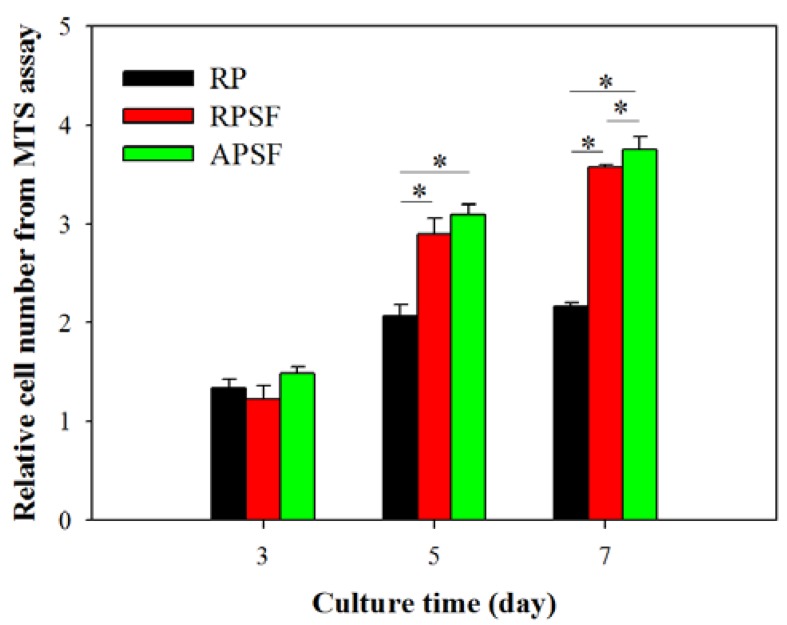
The proliferation of dermal fibroblasts in random PCL (RP), random PCL/SF (RPSF) and aligned PCL/SF (APSF) nanofiber scaffolds. The cell number in each group was normalized to its cell number at day 0. * *p* < 0.05.

Cell proliferation was compared by determining the viable cell number from 3-(4,5-dimethylthiazol-2-y1)-5-(3-carboxymethoxypheny1)-2-(4-sulfopheny1)-2H-tetrazolium (MTS) assays at different time points (day 3, 5 and 7) and normalized to the cell number of each scaffold at day 0 (Figure 4). All scaffolds have a similar relative cell number on day 3, with no significant difference. Although RPSF and APSF have no significant difference in cell number on day 5, both groups have a significantly higher relative cell number compared with RP on day 5 and 7. SF in composite nanofiber scaffolds were shown to promote cell proliferation for chondrocytes, osteoblasts and mesenchymal stem cells [34,35]. Furthermore, an additional contribution from blending PCL with SF may be the enhancement of the hydrophilicity of the nanofiber scaffold, as a balance between the hydrophilicity and hydrophobicity of a scaffold was reported to exert positive effects on cell growth [36]. The introduction of biological functional groups, such as –NH_2_ and –COOH, via SF in RPSF and APSF may also enhance cell proliferation as they were shown to promote cell-scaffold recognition and subsequent proliferation [37]. Considering the effect of fiber alignment, APSF showed no significant difference in relative cell number from RPSF on day 5, but showed significantly higher relative cell number on day 7. Indeed, the physical cue from fiber alignment may further contribute to promote cell proliferation.

#### 2.2.2. SEM Observation

**Figure 5:**
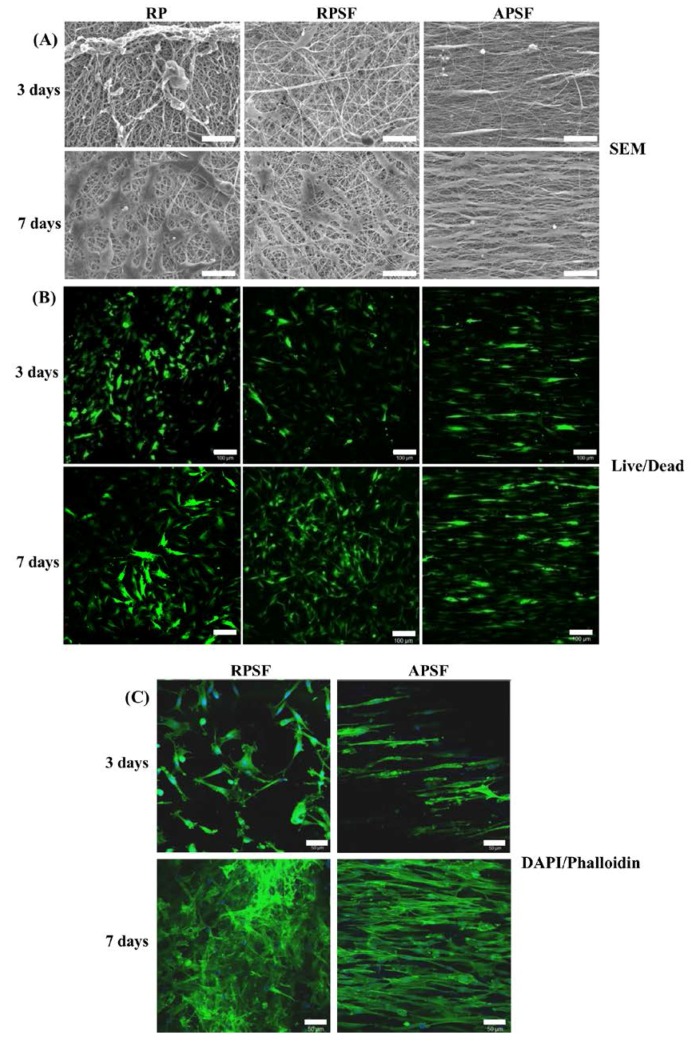
The SEM observation ((**A**), bar = 50 μm), live/dead staining ((**B**), bar = 100 μm) and 4′,6-diamidino-2-phenylindole (DAPI)/phalloidin staining ((**C**), bar = 50 μm) of dermal fibroblasts in random PCL/SF (RPSF) and aligned PCL/SF (APSF) nanofiber scaffolds.

Figure 5A shows the morphology of RDFBs adhered on RP, RPSF, and APSF after cultured for 3 and 7 days. The SEM micrographs on day 3 indicate that cells attached to RP scaffolds had a lower degree of spreading with hindered cytoskeletal extensions compared with RPSF, which is consistent with previous reports that electrospun SF nanofibers could promote keratinocytes and fibroblast adhesion and spreading [38,39]. However, the morphology of attached cells on APSF was significantly different, as the aligned structural arrangement of APSF induced cells to migrate and proliferate in the direction of the nanofiber. On day 7, in which the cells completely attached themselves to the nanofibers, the filopodial extensions were much more visible and higher compared to day 3, irrespective of the random or aligned fiber morphology. The cell distribution on RP and RPSF was more scattered, whereas that on APSF was more aligned with a higher cell density, which is consistent with the viable cell number assay from Figure 4. Based on SEM observation, we conclude that the aligned structural arrangement of APSF aided the oriented growth of RDFBs and provided a favorable environment for cell attachment and proliferation.

#### 2.2.3. Live/Dead Assay

The viability and morphology of cells seeded on random and aligned scaffolds were further examined through live/dead staining. Figure 5B shows the confocal fluorescent micrographs of RDFBs adhered on RP, RPSF, and APSF at day 3 and 7. The live cells (green color) seeded on RP and RPSF were randomly oriented with irregular shapes and minimum dead cells (red color) were found. In contrast, the cells seeded on APSF were oriented in the direction of fiber alignment with elongated shapes with no dead cells observed. At day 7, the cell morphology was similar to what was observed at day 3, but with a significantly higher cell density due to cell proliferation. For RP, a relatively high number of dead cells were observed on day 7, which was consistent with its lower cell proliferation rate compared to RPSF and APSF (Figure 4). Similar to day 3, the cells in APSF proliferated in the direction of fiber alignment, as observed from SEM images (Figure 5A). 

#### 2.2.4. 4′,6-Diamidino-2-Phenylindole (DAPI)/Phalloidin Staining

In order to test the responses of RPSFs to nanofiber scaffolds, cells were cultured in the scaffolds and double stained with fluorescein isothiocyanate (FITC)–phalloidin and DAPI to elucidate the cytoskeletal arrangement of fibroblasts (Figure 5C). Since RP had comparatively less cell spreading, only RPSF and APSF were taken for cytoskeletal staining. On day 3, cells in RPSF exhibited a disorganized actin cytoskeleton in contrast to a cytoskeleton consisting of a large number of actin filaments aligned parallel to the axes of the nanofibers for cells in APSF. The difference in cell morphology was even more pronounced on day 7. Cells were randomly distributed in RPSF to form a thick cell layer, while cells in APSF exhibited a high degree organization of spindle-shaped cytoskeletons, supporting the results observed from SEM and live/dead assays [21]. Hence the results from the DAPI/phalloidin fluorescent staining cross-confirmed that the oriented cell proliferation was solely affected by the nature of the substrate structure originating from its fiber orientation.

#### 2.2.5. Gene Expression

**Figure 6:**
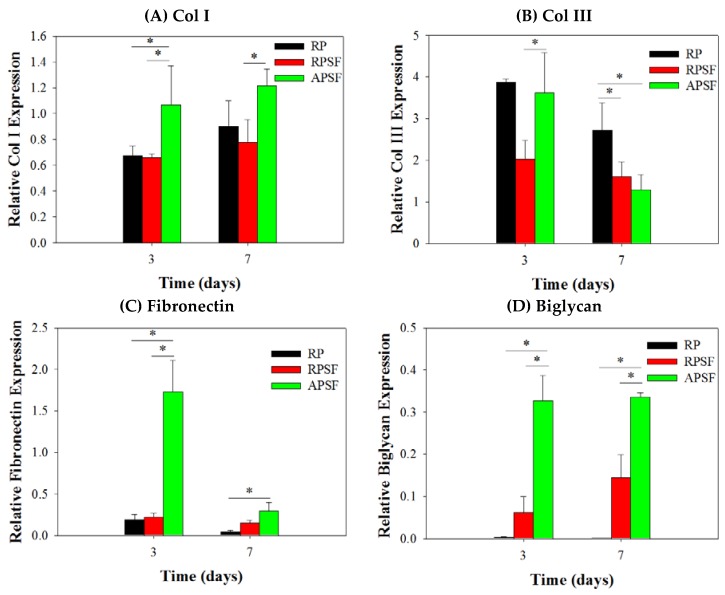
Gene expression of (**A**) type I collagen (Col I); (**B**) type III collagen (Col III); (**C**) fibronectin and (**D**) biglycan in random PCL (RP), random PCL/SF (RPSF) and aligned PCL/SF (APSF) nanofiber scaffolds by quantitative real-time polymerase chain reaction (qRT-PCR). * *p* < 0.05.

The gene expression of tendon marker proteins Col I, Col III, fibronectin and biglycan at three and seven days is shown in Figure 6. In accordance with the nature of early or late expressed genes, Col III and fibronectin showed earlier gene up-regulation (day 3) than Col I and biglycan (day 7). Col I is a crucial protein during the tendon reconstruction process. As collagen matures, the amount of Col I secretion increases to make it a late-stage gene marker for tendon. Higher amounts of Col I indicates an increased number of organized fibers. No significant difference in Col I gene expression was found between RP and RPSF; however, APSF showed significantly higher Col I gene expression than RP and RPSF. This suggests that fiber alignment is the most important factor in inducing the secretion of Col I. Col III is a crucial protein during the period of tendon inflammation and proliferation and is an early-stage gene marker for tendon, as well as the main component of collagen during the early stages of tendon maturation. It will be gradually replaced by longitudinally-aligned long Col I fibrils when the tendon remodels and matures during healing [40]. No significant differences in Col III gene expression were observed between RP and APSF on day 3; however, RPSF and APSF showed significant differences at the same time. This again endorses the advantage of aligned structural arrangement in the production of early marker Col III. Surprisingly, by day 7, RP showed a significantly higher level of Col III expression compared to APSF and RPSF. In other words, the amount of Col III production was higher for cells in RP but not different for cells in RPSF and APSF. Since Col III is produced during the collagen maturation process, we assume that this might be due to the slow collagen maturation process for RDFBs in RP. Moreover, the aligned morphology of APSF could lead to faster tendon reconstruction; thus, cells in APSF show the lowest Col III gene expression.

Fibronectin regulates initial cell attachment and survival and is a well-known marker for active connective tissue repair [41]. Hence, fibronectin is an early-stage marker with its maximum secretion during the cell proliferation stage, which diminishes during the cell maturation stage. The down-regulation of fibronectin gene at day 7 is consistent with other studies, where cells synthesized fibronectin during proliferation and early differentiation. Once the cells reached maturation and accumulated collagens, the production of fibronectin sharply reduced [42]. Significant differences were observed in fibronectin gene expression between RP and APSF as well as APSF and RPSF at day 3. The drastic up-regulation of fibronectin gene expression in APSF could be due to the effect of aligned fiber morphology, which enhanced cell proliferation and increased the cell survival rate. However, at day 7, all scaffolds showed decreased fibronectin gene expression compared to day 3. This indicates cell maturation changes consistent with the trend observed for changes in Col I and Col III production at different times. Biglycan is a glycoprotein, its primary functions being the regulation of growth factor activity during the tendon repair and development process as well as the regulation of the organization, arrangement and the diameter of collagen fibrils in the ECM of the tendon [43]. Biglycan is over-expressed during the re-organization phase of tendon development and repair, coinciding with Col I over-expression. The results confirm that biglycan expression of cells in APSF was the highest among all scaffolds at both time points. This demonstrates the advantage of APSF in facilitating cell organization and arrangements to generate a neo-tendon tissue similar to a native tendon in vitro.

Overall, the qRT-PCR results demonstrated a gene expression profile associated with tendon differentiation in vitro. Furthermore, the up-regulation of tendon matrix protein genes (Col I, fibronectin and biglycan) in RDFBs and tendon reconstruction (Col III) could be shown by combining cues from SF and fiber alignment. A significantly higher gene up-regulation was observed in the aligned composite scaffold (APSF), suggesting a synergistic effect of SF and fiber alignment on RSFBs for tendon tissue engineering in vivo.

### 2.3. Animal Study

#### 2.3.1. Histological Staining

**Figure 7:**
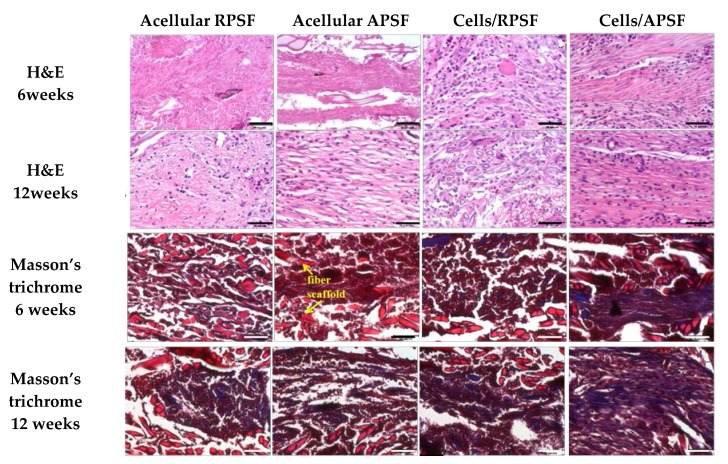
Tissue section staining using hematoxylin-eosin (H&E) and Masson’s trichrome after 6 and 12 weeks. RPSF: random PCL/SF nanofiber scaffolds. APSF: aligned PCL/SF nanofiber scaffolds. Bar = 50 μm.

Figure 7 is the histological staining of explants after 6 and 12 weeks. No cells could be found for acellular scaffolds at week 6 but infiltrated cells from surrounding tissues could be identified at week 12. For cell-seeded scaffolds, both RPSF and APSF exhibited much higher cellular densities across the scaffolds than their acellular counterparts. Though the cell densities were similar, cell orientation was significantly different between the cell-seeded scaffolds. Cell alignment was observed at 6 and 12 weeks in APSF, suggesting that migration and proliferation of RDFBs on aligned nanofibers tend to stretch in a parallel direction along the alignment of the nanofibers. We were able to confirm that the aligned structural arrangement can easily mimic the natural tendon structure to guide RDFBs in vivo. Masson’s trichrome staining was used to evaluate the collagen distribution in ECM of neo-tendon tissues. Since tendons are composed of collagen fibers, such staining methods can clearly depict the extent of collagen deposition and thereby can confirm the formation of neo-tendon tissues. From Masson’s trichrome staining, no significant staining intensity was observed for the absence of collagen fibers in acellular RPSF group at both time points. A similar trend was also observed for the acellular APSF group. Significantly higher staining intensities were observed in cell-seeded implants with a slightly increasing dark blue color intensity of stained collagen. Collagen fibers were randomly oriented in the cells/RPSF group, whereas an oriented distribution was found in the cells/APSF group with more collagen deposition. Thus, Masson’s trichrome staining confirms that the amount and the orientation of ECM deposition of RDFBs is also influenced by the alignment of nanofibers; an advantage offered by cells/APSF constructs in simulating natural tendon environment in vivo.

#### 2.3.2. Immunohistochemical (IHC) Staining

**Figure 8:**
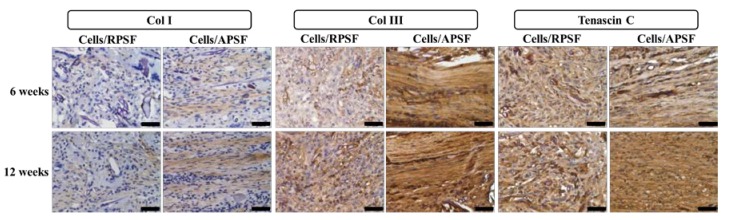
The immunohistochemical staining of type I collagen (Col I), type III collagen (Col III) and tenascin C after 6 and 12 weeks. RPSF: random PCL/SF nanofiber scaffolds. APSF: aligned PCL/SF nanofiber scaffolds. Bar = 50 μm.

The presence of Col I, Col III and tenascin C were confirmed through IHC staining of cell-seeded scaffolds (Figure 8). A general observation of the IHC staining results confirmed Col I, Col III and tenascin C were organized along the direction of fiber alignment in APSF, mimicking native tendon tissue structure. The IHC staining revealed a denser deposition of Col I and Col III in the ECM on aligned nanofibers compared to those on random nanofibers six weeks post-implantation. Furthermore, a denser deposition of another tendon-specific ECM protein, tenascin C, was also evident in APSF. 

Col I is the predominant protein in tendon, making up 86% of the dry weight of the tissue [44]. It is secreted by cells at maturation and evaluation of its presence in implanted samples could confirm tissue reconstruction. At week 6, Col I secreted by cells in RPSF was minimal in contrast to higher Col I production in APSF. At week 12, the deposition of Col I in APSF increased and the distribution became more aligned along the direction of cell distribution. Although Col I also showed an increasing trend from 6 to 12 weeks in RPSF, its content was much lower in comparison with APSF. This result indicated that aligned nanofiber scaffolds promote Col I production in vivo and is in line with the in vitro gene expression results in Figure 6. The physical cues from electrospun nanofibers was suggested to affect cell behavior and fate, as aligned nanofibers induced ligament fibroblast alignment and Col I production [45].

Col III is typically over-expressed during the early inflammatory and proliferative stages of tendon healing. It is characterized by short, disorganized fibrils [7]. The production rate of Col III decreased with the maturation of cells and growth of collagen fibers. A similar relationship between the time-dependent increased deposition of Col I and Col III was observed. At week 6, APSF exhibits increased Col III deposition compared with RPSF. At week 12, the deposition of Col III increased from week 6 for RPSF, but no significant difference was observed for APSF. At this stage, cells in APSF had already entered the tendon reconstruction stage and stopped Col III secretion, which is again consistent with in vitro Col III gene expression (Figure 6). Overall, Col III staining confirms aligned scaffolds could promote tendon maturation and provides cues to lead neo-tendon tissues into the reconstruction phase during tendon regeneration. The aligned structural arrangement of Col III in APSF also re-confirms the effect of nanofiber alignment in the reconstruction of tendon tissue network. 

Tenascin C is a tendon ECM protein secreted during the proliferation or reconstruction of tendons and modulates cell-ECM interactions such as cell adhesion and migration [46]. By week 6, the APSF group had a higher amount of tenascin C than RPSF. This demonstrates the priority of the aligned structure in inducing differentiation of RDFBs to tendon cells, which increased tenascin C secretion. By week 12, the amount of tenascin C in APSF increased significantly and the structural arrangement became more oriented. Conversely, RPSF exhibited no significant changes in tenascin C intensity from week 6. Although the lack of quantitative IHC data may be a limitation of this study, the qualitative IHC results endorse the importance of APSF in inducing oriented arrangement of cells and differentiation of RDFBs to tenocytes. The IHC results thus confirmed the advantage of uni-axial fiber orientation in the reconstruction of tendon tissues in vivo.

#### 2.3.3. Biomechanical Testing

Structural biomechanical properties (linear stiffness and maximum force) that depend on specimen dimensions were determined for normal tendon and explanted acellular and cell-seeded scaffolds 12 weeks post-operation (Table 2). By 12 weeks post-operation, the tensile stiffness of cells/RPSF and cells/APSF reached 28.4% and 60.2% of that of normal tendon, respectively. The mean tensile stiffness of the cells/RPSF group was significantly lower than that of the cells/APSF group, but both are significantly different from that of normal tendon. The mean tensile stiffness of acellular APSF and RPSF are also significantly less than those of their cell-seeded counterparts. For maximum load, a similar trend was observed for acellular and cell-seeded scaffolds. Hence, the ability of the scaffolds to reach a significant strength was clearly not a result of the acellular scaffold but rather from the regenerated tissue, while the aligned nanofiber scaffold APSF resulted in a significantly higher tensile stiffness or load compared with RPSF. Comparing the cell-seeded scaffolds, the mean ultimate load of cells/RPSF and cells/APSF are significantly different, and the values reached 43.8% and 81.3% of that of normal tendon, respectively. Most importantly, there is no significant difference in maximum load between cells/APSF and normal tendon (*p* > 0.05).

Our biomechanical analysis results compare favorably with those from previous Achilles tendon repair studies. Bone marrow mesenchymal stem cell-collagen gel constructs were implanted in immature rabbits with a 1-cm long gap defect in the Achilles tendon. The acellular and cell-seeded scaffolds respectively gave 32% and 63% in stiffness and 30% and 69% in maximum force as percentages of normal Achilles tendon values [47]. A similar study employing a different cell-to-collagen ratio was used to repair 2-cm gap defects in rabbit Achilles tendons [48]. The best construct gave 53% maximum force and 64% tensile stiffness of normal tendon values at 12 weeks after surgery. For comparison, the cells/APSF group showed a substantially larger maximum load and similar stiffness in our study using the nanofiber scaffold. In a different study, the mean tensile stiffness was reported to be 56% and 87% of normal tendon values for acellular and bone marrow stromal cell-seeded knitted poly-lactide-co-glycolide scaffolds at 12 weeks post-operation in regeneration of rabbit Achilles tendon with 1-cm gap defect [8]. Interesting, although the mean stiffness of regenerated tendon is smaller in our study using RDFBs in APSF, the increase of stiffness from acellular to cell-seeded scaffolds is similar in both studies (31% vs. 32%).

## 3. Materials and Methods

### 3.1. Materials

Polycaprolactone (PCL, average molecular weight = 80,000 Da) was purchased from Sigma-Aldrich (St. Louis, MO, USA). *Bombyx mori* silk fiber was supplied by Shitan Chuanmin silkworm farm in Miaoli County, Taiwan. Fetal bovine serum (FBS) and Dulbecco’s Modified Eagle’s medium (DMEM) were purchased from Thermo Fisher Scientific (Waltham, MA, USA). Dichloromethane (DCM) and dimethylformamide (DMF) were purchased from J.T. Baker (Philipsburg, NJ, USA). Trifluoroacetic acid (TFA) and hexafluoroisopropanol (HFIP) were purchased from Sigma-Aldrich (St. Louis, MO, USA). Dialysis membrane tubes (CelluSep H1) were purchased from Orange Scientific (Braine-l’Alleud, Belgium). Except *Bombyx mori* silk fiber, all chemicals were used as received.

### 3.2. Preparation of Nanofiber Scaffolds by Electrospinning

Silk fibroin (SF) was prepared from *B. mori* silk by a stepwise purification method [49]. Electrospinning was adopted for the preparation of random PCL (RP) nanofibers, random PCL/SF (RPSF) nanofibers and aligned PCL/SF (APSF) nanofibers. PCL was dissolved in a DCM/DMF (4:1, *v*/*v*) solvent mixture to prepare a 10% (*w*/*v*) PCL solution. SF and PCL were dissolved in a TFA/ HFIP (1:3, *v*/*v*) solvent mixture to obtain a SF/PCL solution having a final concentration of 26% (*w*/*v*) (13% SF and 13% PCL). Five milliliters of pre-made electrospinning solution of PCL and SF/PCL was pumped from a syringe fitted with a 23-gauge needle at 1 mL/h and 30 kV. The fibers were collected by a grounded static collector kept 15 cm from the tip of the needle to obtain RP and RPSF nanofibers, respectively, with ~100 μm thickness. The APSF nanofibers were prepared with the same thickness using the same electrospinning setup but at 16 kV voltage and using a rotating collector (10 cm diameter, rotate at 3500 rpm) and 10 cm tip-to-collector distance. The thickness of all scaffolds was measured with a dial thickness gauge (TECLOCK SM-1201, Nagano, Japan). All SF-containing nanofibers were immersed in a 7% (*v*/*v*) ammonia/75% (*v*/*v*) ethanol solution at room temperature for 30 min to remove residual TFA and to attain the water insolubility of SF [24]. Subsequently, the membrane was washed with copious phosphate buffered saline (PBS) to remove ammonia. The membranes were dried in an oven at 37 °C before use.

### 3.3. Characterization of Nanofiber Scaffolds

#### 3.3.1. SEM Analysis

The prepared fibrous membranes were cut into 0.5 cm × 0.5 cm square pieces and pasted onto a carbon tape fixed aluminum stub for conductive coating at 20 mA for 60 s. The morphology of nanofibers was examined with a scanning electron microscope (SEM) (S-3000N, Hitachi Ltd., Tokyo, Japan) and the average fiber diameter was determined using ImageJ software from at least 100 fibers from 10 images. The orientation of nanofibers was calculated from the frequency of fibers with fiber orientation (degree) ranging from −90° to 90° relative to a defined vertical direction (0°) from 100 fibers. The fiber orientation was analyzed using circular statistics with the CircStat toolbox for MATLAB (MathWorks, Natick, MA, USA) to calculate the angular distribution value.

#### 3.3.2. TGA and DTA

A thermogravimetric analysis (TGA) and derivative thermogravimetric analysis (DTA) were performed to evaluate the thermal properties of RP and RPSF nanofibers in nitrogen atmosphere. Samples were allowed to undergo thermal decomposition at a controlled heating rate of 10 °C/min from 25 to 500 °C using TGA 2050 from TA instruments (New Castle, DE, USA). The decomposition behavior was monitored by plotting weight (%) and derivative weight (%/°C) vs. temperature (°C). 

#### 3.3.3. XRD and FTIR Analysis

The X-ray diffraction (XRD) patterns were obtained from a D5005 X-ray diffractometer (Siemens AG, Munich, Germany) from 5° to 60° at a scanning speed of 1.2° min^−1^. Fourier-transform infrared (FTIR) spectroscopy was used for chemical analysis using a Horiba FT-730 spectrometer (Horiba, Ltd., Kyoto, Japan) in the attenuated total reflection configuration. The scanning wavenumber was from 600 to 2000 cm^−1^ with a resolution of 2 cm^−1^.

#### 3.3.4. Mechanical Testing

Tensile properties were evaluated using a H1KT tabletop mechanical testing machine (Tinius Olsen, Horsham, PA, USA) having a 10 N loading cell. Samples were cut into strips (10 × 50 mm^2^) and allowed to undergo tensile elongation at a crosshead speed of 5 mm/min and a gauge length of 30 mm. Young’s modulus (MPa), ultimate stress (MPa) and ultimate strain (%) were calculated from the tensile stress-strain curves (*n* = 6).

### 3.4. In Vitro Cell Culture

#### 3.4.1. Isolation of Rabbit Dermal Fibroblasts (RDFBs)

Fresh skin harvested from the back of New Zealand white rabbits (2–3 kg weight) were collected under aseptic condition. After thorough washing in PBS and soaking in 0.25% chloramphenicol solution for 10 min, the harvested skins were minced into small pieces (2 × 2 × 2 mm^3^) and subjected to digestion with 0.25% type I collagenase in DMEM high glucose at 37 °C in a rotation incubator for 8 h. The isolated cells were plated on T75 flask and cultured in DMEM containing 10% FBS, penicillin (100 U/mL), streptomycin (100 μg/mL), and ascorbic acid (50 μg/mL) at 37 °C in a CO_2_ incubator with medium change every three days. Cells from the second and third passage were used for the in vitro studies.

#### 3.4.2. Cell Proliferation

All nanofiber scaffolds were treated with 75% ethanol overnight and rinsed twice with PBS for 30 min before use. The membrane (15 mm diameter) was pre-conditioned by immersion in DMEM for 1 day and placed in a 24-well culture plate and each well was seeded with 100 μL of RDFBs cell suspension (5 × 10^4^ cells) and incubated at 37 °C for 4 h to allow cell adhesion. The membrane was transferred to a new well and cultured in 1.5 mL cell culture medium (DMEM supplemented with 10% FBS and 1% antibiotic-antimycotic) in a CO_2_ incubator at 37 °C with medium change every 2 days. The number of viable cells was determined at day 3, 5 and 7 using MTS assays with the CellTiter 96^®^ AQueous One Solution from Promega (Madison, WI, USA). An ELISA plate reader (Synergy HT, BioTek, Winooski, VT, USA) was used for colorimetric measurements of the formazan product at 492 nm.

#### 3.4.3. SEM Observation

For SEM observations, cell-seeded scaffolds at day 3 and 7 were fixed in 2.5% glutaraldehyde solution and dehydrated through a graded series of ethanol soaks, and finally dried using hexamethyldisilazane overnight. Completely dehydrated samples were sputter-coated with gold at 20 mA for 60 s. Samples were mounted on aluminum stubs, fixed with carbon tapes and observed under a SEM (Hitachi S-3000N, Hitachi Ltd., Tokyo, Japan, Hitachi S3000N) at an accelerating voltage of 10 kV.

#### 3.4.4. Live/Dead Assay

Cell viability was assessed by using the live/dead viability/cytotoxicity assay kit from Thermal Fisher Scientific (Waltham, MA, USA), which allows the simultaneous fluorescence staining of viable and dead cells. RDFBs were cultured in nanofiber scaffolds for 3 and 7 days, followed by removal of the medium and PBS washing for three times. The solution for Live/Dead cell staining was prepared by mixing 3 μL of 4 mM calcein AM solution (for live cells) (excitation at 494 nm and emission at 517 nm) and 5 μL of 2 mM ethidium homodimer-1 (EthD-1) solution (for dead cells) (excitation at 528 nm and emission at 617 nm) in 10 mL cell culture medium [21]. One milliliter of staining solution was added to each cell-seeded scaffold placed in a well of a 24-well culture plate in dark. The scaffold was incubated at 37 °C for 30 min, followed by observation under a confocal laser scanning microscope (Zeiss LSM 510 Meta, Oberkochen, Germany). 

#### 3.4.5. DAPI/Phalloidin for Cytoskeletal Staining

The cytoskeleton arrangements of attached RDFBs on nanofiber scaffolds were determined from F-actin staining at day 3 and 7. The scaffolds were fixed in 4% paraformaldehyde for 10 min after washed with PBS. The scaffolds were further washed repeatedly in PBS and treated with 0.1% Triton X-100 in PBS for 10 min. After washing twice with PBS, the samples are stained with 20 μg/mL fluorescein isothiocyanate (FITC)-phalloidin for 30 min. After additional washing in PBS and staining cell nuclei were stained with 1 μg/mL (4′,6-diamidino-2-phenylindole) (DAPI) for 5 min, the samples were observed under a confocal laser scanning microscope (Zeiss LSM 510 Meta, Oberkochen, Germany), The excitation and emission wavelengths for DAPI are 358 and 461 nm, respectively, and the corresponding wavelengths for FITC-phalloidin are 496 and 516 nm.

#### 3.4.6. RNA Extraction and cDNA Synthesis

The RNA isolation and cDNA synthesis steps were performed as per standard procedures by using TRIzol from Invitrogen (Carlsbad, CA, USA) to isolate RNA from RDFBs. Cell suspension was collected by rupturing the membranes and the solution obtained was placed in a 1.5 mL micro centrifuge tube. 

After adding 200 μL chloroform, the solution was vortexed for 15~30 s, followed by incubation in an ice bath for 5 min and centrifuged at 12,000× *g* for 15 min. The supernatant RNA layer was isolated and reacted with an equal volume of isopropanol at −80 °C for 30 min. The solution was further centrifuged at 12,000× *g* for 15 min at 4 °C. The supernatant was removed and 1 mL of 75% ice cold ethanol was added. The solution was mixed at 4 °C for 10 min and centrifuged at 12,000× *g* for 10 min. This step was repeated twice and the final supernatant solution was removed. The precipitate was dried at room temperature for 10 min and incubated with 20 μL of DEPC (Invitrogen, Carlsbad, CA, USA) treated water at 55–60 °C for 30 min for complete dissolution of RNA. 

#### 3.4.7. Quantitative Real-Time Polymerase Chain Reaction (qRT-PCR)

SuperScript III reverse transcriptase (Invitrogen, Carlsbad, CA, USA) was used to reversely transcribe total RNA into cDNA. Glyceraldehyde 3-phosphate dehydrogenase (GAPDH) was used as the reference gene for internal control. A CFD-3120 Mini Option detection system (Bio-Rad, Hercules, CA, USA) together with SYBR Green RT-PCR kit (SYBR Green I SuperMix, Bio-Rad Laboratories Inc., Hercules, CA, USA) were used for the qRT-PCR measurements using the 2^−ΔΔCT^ relative quantification method. Gene expression of type I collagen (Col I), type III collagen (Col III), fibronectin and biglycan were analyzed with the primer sequences reported before (*n* = 6) [7].

### 3.5. Animal Study

#### 3.5.1. Implant Preparation and Experimental Design

One hundred microliter of RDFBs cell suspension (2.5 × 10^5^ cells) were seeded evenly onto sterilized RPSF or APSF nanofiber scaffolds (30 mm × 30 mm × 0.1 mm) in culture dishes and cultured in a CO_2_ incubator for one week with medium change every 2 days to form cell-scaffold constructs. Forty-eight 6-month-old New Zealand white rabbits (National Laboratory Animal Breeding and Research Center, Taipei, Taiwan, China) were used in this study, as per the guidelines of the Institutional Animal Care and Use Committee of Chang Gung University (IACUC Approval No.: CGU14-139). Experimental animals were randomly divided into 4 groups, including acellular RPFS, acellular APFS, cells/RPSF and cells/APFS (*n* = 12 in each group). Each group was then divided into two subgroups of 6 and 12 weeks post-operation (*n* = 6). An Achilles tendon partial defect model of the posterior leg was designed and the rabbits were pre-anesthetized by the intramuscular injection of ketamine (20 mg/kg). The posterior leg of rabbits was carefully shaved and general anesthesia was then induced by 4% isoflurane and was maintained with 2% isoflurane with O_2_ at 2.5 L/min throughout the surgery. The rabbit was put in a prone position with full ankle extension and the surgical field of hind leg was sterilized. All surgical instruments were sterilized before the operation and were kept sterile during the whole procedure. Through a 5-cm longitudinal incision at the left posterior leg, a 2-cm lateral segment of the Achilles tendon with paratenon was excised and the medial half was left intact. For each of the experimental group, the cell-seeded scaffold was placed on top of a SF microfibrous membrane of the same size (30 mm × 30 mm × 0.2 mm), wrapped and tied to form a cord-shape implant (3-mm diameter) that mimicked the tendon size. The implants were inserted into the defect and a modified Kessler core pattern suture was anchored at both ends of the remaining tendon by using a 4-0 polydioxanone suture (PDS) [50]. The implantation of acellular scaffolds without RDFBs was performed in the same way as a control. After surgery, the skins were closed by 4-0 Ethilon^®^ sutures (Ethicon, Johnson & Johnson, New Brunswick, NJ, USA) and 3 mg/kg gentamicin was administered intramuscularly as prophylactic antibiotics. The wounds were dressed by gentamicin ointment to prevent infection and rabbits were kept in cages for their free activities. The detailed surgical procedure is provided in the Supplementary Materials. 

#### 3.5.2. Histological Observation

At 6 and 12 weeks, six rabbits from each group were euthanized with lethal doses of pentobarbital (0.5 g/kg bodyweight). The specimens were harvested and subject to histological examination of 4 μm slices of 10% formaldehyde-fixed and paraffin-embedded samples. Hematoxylin and eosin (H&E), Masson’s trichrome and immunohistochemical (IHC) staining of Col I, Col III and tenascin C were performed following standard protocols [51]. 

#### 3.5.3. Biomechanical Tests

The mechanical features of 12-week implanted samples were investigated through biomechanical testing within 6 h of explantation. A universal testing machine (UN-7001, Gotech Testing Machines Inc., Taichung City, Taiwan) with a 5000 N load cell was used by fixing each end of the harvested sample to a non-slip clamp (TA-2) and elongated axially at a speed of 2 mm/min until rupture. Structural properties were determined from the load-elongation curves, including the maximum load (N) at failure and the tensile stiffness (N/mm) that was calculated from the linear region of the force-displacement curve (*n* = 6).

### 3.6. Statistical Analyses

All data were expressed as the mean ± standard deviation (SD). A one-way ANOVA LSD test was used for statistical analyses with a *p* value less than 0.05 considered statistically significant.

## 4. Conclusions

We have demonstrated the effects of SF and fiber alignment in PCL-based nanofibers on scaffold physicochemical properties, cell response in vitro and tendon tissue regeneration in vivo in this study. A well-dispersed blending of SF with PCL could be confirmed and electrospinning was able to produce nanofiber scaffolds with random and aligned nanofibers. The aligned composite APSF scaffold exhibited the highest Young’s modulus and hydrophilicity. In vitro cell culture studies confirmed that the addition of SF favored cell proliferation. SEM and confocal microscopic analysis confirmed the effect of fiber alignment on the direction of cell migration and proliferation. Overall, the APSF scaffold facilitated cell proliferation, up-regulated gene expression of tendon-specific ECM proteins in vitro and increased the production and deposition of collagen and tenascin C in vivo, indicating its potential in tendon tissue engineering applications. The nanofiber scaffold developed in this study demonstrates the combined effect of biochemical and physical cues to ameliorate the biological and mechanical properties of the neo-tendon tissue for repair of Achilles tendon defects in rabbit/animal model.

## Figures and Tables

**Table 1:** Mechanical properties of random PCL (RP), random PCL/SF (RPSF) and aligned PCL/SF (APSF) nanofiber scaffolds. Values are mean ± SD of six independent measurements.

Scaffold	Ultimate Stress (MPa)	Ultimate Strain (%)	Young’s Modulus (MPa)
RP	1.48 ± 0.21	39.75 ± 6.61	15.28 ± 5.31
RPSF	0.06 ± 0.01 *	2.05 ± 0.30 *	3.93 ± 0.05 *
APSF	0.94 ± 0.11 *^,#^	1.72 ± 0.19 *	70.52 ± 2.83 *^,#^

Values are means ± SD. * *p* < 0.05 compared with RP; ^#^ *p* < 0.05 compared with RPSF. PR: random PCL nanofiber scaffolds. RPSF: random PCL/SF nanofiber scaffolds. APSF: aligned PCL/SF nanofiber scaffolds.

**Table 2:** Biomechanical tests of normal tendon, regenerated tendons from acellular RPSF, acellular RPSF, cells/RPSF and cells/APSF after 12 weeks. Values are mean ± SD of six independent measurements.

Groups	Stiffness (N/mm)	Maximum Load (N)
Normal tendon	29.9 ± 5.8	382.3 ± 58.2
Acellular RPSF	4.5 ± 0.7	114.3 ± 11.6
Acellular APSF	10.1 ± 1.8	216.4 ± 33.0
Cells/RPSF	8.5 ± 1.1	167.3 ± 25.4
Cells/APSF	18.0 ± 3.8 *	310.9 ± 73.5 *^,#^

Values are means ± SD. * *p* < 0.05 compared with cells/RPSF; ^#^ *p* > 0.05 compared with normal tendon. RPSF: random PCL/SF nanofiber scaffolds. APSF: aligned PCL/SF nanofiber scaffolds.

## Supplementary Materials

The following are available online at www.mdpi.com/2079-4991/7/8/219/s1, Figure S1: Surgical procedure for repairing Achilles tendon defect repairs in rabbits with RPSF and APSF scaffolds. (A) The electrospun scaffold (right) seeded with dermal fibroblast cells was wrapped to itself with seal off two ends; (B) exploration of rabbit Achilles tendon; (C) isolation of the lateral segment of the Achilles tendon; (D) a 2-cm tendon defect was made completely; (E) the defect was repaired with the cells/scaffold construct using modified Kessler suture technique; (F) gross view immediate after tendon repair.
