# Supplementary material for: Response of Dermal Fibroblasts to Biochemical and Physical Cues in Aligned Polycaprolactone/Silk Fibroin Nanofiber Scaffolds for Application in Tendon Tissue Engineering
Source: Nanomaterials (Basel). 2017 Aug 11;7(8):219. doi: 10.3390/nano7080219 (PMC5575701; doi:10.3390/nano7080219)
Supplement: Supplementary file 1 [file nanomaterials-07-00219-s001.pdf]

Supplementary Material

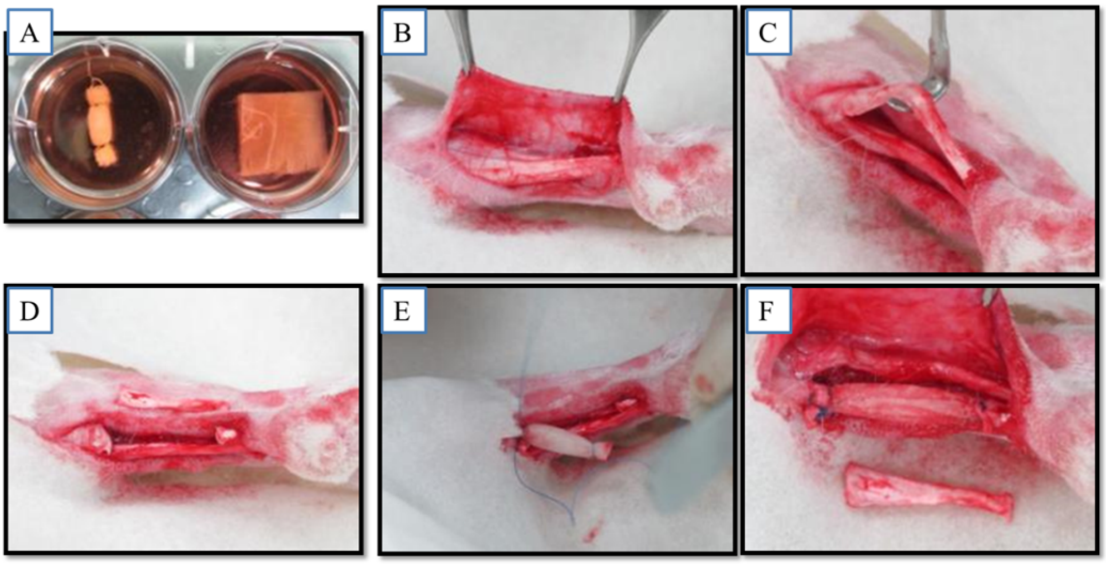

Surgical procedure for repairing Achilles tendon defect repairs in rabbits with RPSF and APSF scaffolds. (A) The electrospun scaffold (right) seeded with dermal fibroblast cells was wrapped to itself with seal off two ends; (B) exploration of rabbit Achilles tendon; (C) isolation of the lateral segment of the Achilles tendon; (D) a 2-cm tendon defect was made completely; (E) the defect was repaired with the cells/scaffold construct using modified Kessler suture technique; (F) gross view immediate after tendon repair.
